# Dietary Supplementation Impact of Combined Broccoli and Citrus Peel By‐Products on the Growth Performance of Japanese Abalone (*Haliotis discus*, Reeve 1846) and Stress Resistance to Various Stressors

**DOI:** 10.1155/anu/7880258

**Published:** 2026-01-03

**Authors:** Ran Li, Sung Hwoan Cho

**Affiliations:** ^1^ College of Xingzhi, Zhejiang Normal University, Jinhua, 321004, China, zjnu.edu.cn; ^2^ Division of Convergence of Marine Science, National Korea Maritime and Ocean University, Busan, 49112, Republic of Korea, kmou.ac.kr

**Keywords:** biocompounds, combination of broccoli and citrus peel by-product (CBP), functional additive, *Haliotis discus*, immune response, stress reducer

## Abstract

This experiment was conducted to investigate the impacts of dietary incorporation of a combination of broccoli by‐product and citrus peel by‐product (CBC) as a functional additive on the growth and resistance of the Japanese abalone (*Haliotis discus*) under various stressor conditions. A total of 2520 abalone juveniles (initial weight of 3.33 g) were assigned to 21 net cages, with 120 individuals per cage and three cages per feed. Six formulated feeds, supplemented with 0%, 0.5%, 1%, 2%, 3%, and 5% CBC, were prepared and labeled as the control (Con), CBC0.5, CBC1, CBC2, CBC3, and CBC5 feeds, respectively. Additionally, dry *Saccharina japonica* was prepared to compare the performance of abalone fed with the formulated feeds. All abalone were fed once daily for 16 weeks. Following the 16‐week feeding experiment, 60 abalone from each cage were randomly chosen and evenly divided into 3 groups. These groups were then exposed to stressors: 20 h air exposure, 20 h high temperature exposure (30°C), and 12 h low salinity exposure (25 psu). The survival of abalone was checked for 5 days following these stress tests. The specific growth rate (SGR) of abalone fed all artificial feeds was statistically (*p* < 0.05) greater than that of abalone fed the *S. japonica*, but no statistical differences were observed among abalone fed the different formulated feeds. The shell length, width, and height, and soft body weight of abalone fed with all artificial feeds were statistically superior (*p* < 0.05 for all) compared to those fed with *S. japonica*. After the 5‐day observation period following 20 h air exposure or high temperature exposure at 30°C, the survival rates of abalone fed with CBC2, CBC3, and CBC5 feeds were statistically (*p* < 0.05) greater than those fed with *S. japonica*. Increasing the CBC inclusion level (0–5%) in feeds linearly enhanced the abalone survival under 20 h air and high temperature exposures. Therefore, CBC exhibited great potential as a stress reducer in abalone feed, and the inclusion of at least 2% CBC in formulated feeds is recommended to enhance abalone’s resistance to air and high‐temperature stressors.

## 1. Introduction

Abalone (*Haliotis* spp.) is a widely distributed gastropod that lives in both tropical and temperate waters around the world [1, 2]. The global demand for abalone is continuously increasing in the market due to its high nutrients, desirable taste, and considerable medicinal values [3–5]. However, the fishery production of abalone has declined because of the dramatic environmental changes and degradation of marine fishery resources [1]. The global production from legal abalone fisheries has reduced from around 20,000 metric tons (MT) in the 1970s to barely about 4500 MT in 2020, whereas the production of farmed abalone has elevated from a tiny amount in 1970s to more than 240,000 MT in 2020 [6]. Commercial abalone farmers preferred feeding abalone with live or dried macroalgae (MA), such as *Saccharina japonica*, *Gracilaria* spp., *Laminaria japonica*, and *Undaria pinnatifida* [7–9]. On the other hand, owing to the insufficient nutrient density, limited seasonal supply, introduction of disease and pests to culture systems, and increasing price of MA, nutrition‐balanced artificial diets have also been developed [8, 9]. Previous studies [3, 10, 11] have demonstrated that abalone fed with formulated feeds exhibited improved growth performance and stronger resistance to various stressors compared to those fed a single MA.

Agriculture by‐products with a stable supply and low cost can be incorporated into abalone feeds. Citrus peel by‐product (CPB) [10, 12], onion extract by‐product [13], cabbage extract by‐product [14], rice bran meal [9, 15], broccoli by‐product (BBP) [3], carrot leaf [16], and beat leaf by‐product [7] have been successfully demonstrated to serve as the replacers for MA or as additives in abalone diets. For instance, Li et al. [3] highlighted that BBP could be applied as an excellent substitute for *S. japonica* in abalone diets, with a 25% substitution of BBP for *S. japonica* resulting in the most outstanding growth performance. Furthermore, the best growth performance and highest survival rates under air exposure and high temperatures were noticed in abalone (*H. discus*) given a diet enriched with 5% CPB when abalone were provided with one of the formulated feeds comprising 0.5%, 1%, 2%, 3%, and 5% CPB for 16 weeks, followed by being exposed to air, low salinity, and high temperature [10]. They concluded that CBP can be considered as an effective growth enhancer and stress reducer in abalone feed. According to Yun et al. [12], *U. pinnatifida* could be completely substituted by CPB in formulated diets without hindering the growth performance of abalone or reducing their resistance to air exposure stress, and substituting 50% of *U. pinnatifida* with CPB in diets resulted in the best weight gain for abalone. Since broccoli and citrus are abundant in flavonoids, phenolic substances, and vitamins [3, 17–19], their by‐products can also be considered an effective additive in aquafeeds. In addition, the combined bio‐compounds or synthetic chemicals are more effective than the respective single compounds as a functional additive [4, 20], as they can compensate for each other’s nutrient or chemical deficiency.

The stress resistance in abalone is of great significance in aquaculture practice, as environmental stress can compromise their physiological status and lead to reduced production [21]. Several potentially stressful abiotic factors, such as air exposure during grading and transportation, along with fluctuation in temperature, oxygen, and salinity of seawater during the summer season, notably influence growth and survival of abalone [21, 22]. Under oxidative stress‐induced conditions, feeding abalone with feeds supplemented with antioxidants could enhance its antioxidant capacity [11]. However, the use of commercial antioxidants, such as ethoxyquin, to prevent spontaneous oxidation in feeds may lead to residue accumulation in the flesh of aquatic animals, which can subsequently be delivered to humans via consumption [10, 23]. Previous research [24, 25] found that commercial antioxidants might lead to DNA damage and abnormalities in chromosomes in human lymphocytes in vitro. Consequently, feed nutritionists are exploring natural antioxidant additives for aquafeeds. For example, Oniszczuk et al. [26] found that the purple coneflower (*Echinacea purpurea*) has the potential to be used as a natural radical scavenger and nutritive ingredient in fish diets. Li et al. [27] reported that extracts of Chinese angelica (*Angelica sinensis*) could reduce lipid oxidation in fish feed, with high concentrations showing significant inhibitory effects. Additionally, dietary inclusion of ethyl acetate extracts of Chinese angelica at a concentration greater than 2.0 g/kg enhanced growth and feed efficiency of Jian carp (*Cyprinus carpio*).

Because most studies focus on evaluating a single replacer or additive in abalone diets, research on the combined use of additives and their potential synergistic effects remains limited. Given the high substitutability of BBP for *S. japonica* [3] and the beneficial impacts of CPB as an additive in abalone feeds [10], this study was performed to evaluate the effects of including a combination of BBP and CPB (CBC) in diets on the growth, chemical composition of the soft body, and stress resistance of abalone.

## 2. Materials and Methods

### 2.1. Preparation of CBC

BBP and CPB were provided by Dawon Farm and Ilhae Corporation (Jeju Special Self‐Governing Province, Korea), respectively, and then dried at 40°C for 48 h. Once dried, BBP and CPB were ground into powder (≤ 200 µm) and mixed at a ratio of 1:1.

### 2.2. Preparation of Abalone Feeds

Six artificially formulated feeds were designed (Table 1). In the control (Con) diet, 25% fish meal, 7% casein, and 6% defatted soybean meal were included as the main protein sources. Additionally, 20% wheat flour and each of 0.5% squid liver oil and soybean oil were also added to the Con diet as the main carbohydrate and lipid sources, respectively. In the Con diet, 0.5%, 1%, 2%, 3%, and 5% CBC were incorporated at the cost of wheat flour, labeled as the CBC0.5, CBC1, CBC2, CBC3, and CBC5 diets, respectively. Finally, dry *S. japonica* was prepared to compare the growth performance of abalone fed with the formulated diets.

**Table 1:** Ingredients of the experimental feeds (%, dry matter basis).

	Experimental diets						
	Con	CBC0.5	CBC1	CBC2	CBC3	CBC5	*S. japonica*
Ingredient (%)							
Fish meal	25.0	25.0	25.0	25.0	25.0	25.0	—
Casein	7.0	7.0	7.0	7.0	7.0	7.0	—
Defatted soybean meal	6.0	6.0	6.0	6.0	6.0	6.0	—
Wheat flour	20.0	19.5	19.0	18.0	17.0	15.0	—
Combined broccoli by‐product^a^ and citrus peel by‐product^b^ (CBC)	0.0	0.5	1.0	2.0	3.0	5.0	—
* S. japonica* powder	20.0	20.0	20.0	20.0	20.0	20.0	—
Squid liver oil	0.5	0.5	0.5	0.5	0.5	0.5	—
Soybean oil	0.5	0.5	0.5	0.5	0.5	0.5	—
Sodium alginate	18.0	18.0	18.0	18.0	18.0	18.0	—
Mineral premix^c^	2.0	2.0	2.0	2.0	2.0	2.0	—
Vitamin premix^d^	1.0	1.0	1.0	1.0	1.0	1.0	—
Nutrients (%)							
Dry matter	94.6	94.6	94.5	94.7	94.7	94.5	84.9
Crude protein	30.5	30.2	30.4	30.1	30.0	30.3	11.5
Crude lipid	4.4	4.4	4.3	4.4	4.3	4.3	1.1
Ash	9.1	9.2	9.3	9.3	9.5	9.5	17.5
Carbohydrate^e^	56.1	56.2	55.9	56.2	56.2	55.9	69.9

Abbreviations: BBP, broccoli by‐product; CPB, citrus peel by‐product; *S. japonica*, *Saccharina japonica*.

^a^Broccoli by‐product (BBP) was provided from Dawon farm (Jeju Special Self‐Governing Province, Korea).

^b^Citrus peel by‐product (CPB) was provided from Ilhae Corporation (Jeju Special Self‐Governing Province, Korea).

^c^Mineral premix contained the following ingredients (g/kg mix): NaCl, 10; MgSO_4_ · 7H_2_O, 150; NaH_2_PO_4_ · 2H_2_O, 250; KH_2_PO_4_, 320; CaH_4_(PO_4_)_2_ · H_2_O, 200; Ferric citrate, 25; ZnSO_4_ · 7H_2_O,4; Ca‐lactate, 38.5; CuCl, 0.3; AlCl_3_ · 6H_2_O, 0.15; KIO_3_, 0.03; Na_2_Se_2_O_3_, 0.01; MnSO_4_^․^H_2_O, 2; CoCl_2_ · 6H_2_O, 0.1.

^d^Vitamin premix contained the following amount which were diluted in cellulose (g/kg mix): L‐ascorbic acid, 200; *α*‐tocopheryl acetate, 20; thiamin hydrochloride, 5; riboflavin, 8; pyridoxine, 2; niacin, 40; Ca‐D‐pantothenate, 12; myo‐inositol, 200; D‐biotin, 0.4; folic acid, 1.5; p‐amino benzoic acid, 20; K_3_, 4; A, 1.5; D_3_, 0.003; choline chloride, 200; cyanocobalamin, 0.003.

^e^Carbohydrate = 100 – (crude protein + crude lipid + ash).

All prepared components were properly combined with water at a 1:1 ratio. The mixed paste for each diet was formed into sheets 0.15 cm thick and cut into 1 cm × 1 cm slices. These slices were then immersed in a 5% CaCl_2_ aqueous solution for 1 min and dried for 48 h at room temperature. Finally, all prepared slices were kept at −20°C until use. All formulated feeds fulfilled the protein and lipid requirements of abalone [28–31].

### 2.3. Experimental Conditions

The Japanese abalone (*Haliotis discus*) juveniles were obtained from a local farm (Daegun abalone farm, Jeju Special Self‐Governing Province, Korea). Prior to the experiment, abalone were provided with dry *S. japonica* at a biomass ratio of 1.5%–3.0% daily for 14 days. After the 14‐day acclimation period, a total of 2520 abalone (initial weight of 3.33 g) were randomly assigned into 21, 100 L (0.5 m × 0.4 m × 0.5 m) net cages (120 abalone/cage). Three 3.6‐ton flow‐through raceways (water volume: 3.4 tons) were utilized in this study, with seven net cages per raceway. Proper aeration and underground seawater (108 L/min) were continuously provided to each raceway, and photoperiod was kept in its natural state. The water temperature changed from 17.9 to 18.6°C (18.1 ± 0.12°C; mean ± SD). All diets were supplied to triplicate cages of abalone once (17:00) daily for 16 weeks. Net cages were cleaned every other day before feeding, and dead abalone were promptly eliminated when noticed. After the 16‐week experiment, all live abalone were fasted for 24 h.

### 2.4. Water Stability of the Experimental Feeds

10 g of each prepared feed were held in 63 lab dishes, with nine dishes per diet. All dishes were placed into 9, 100 L plastic tanks (7 dishes per tank) at a flow rate of 1.4 L/min without abalone, and then sampled at 12, 24, and 48 h after immersion. The water stability of nutrients in feeds was presented as the percentage difference between the final and initial dry content of each nutrient. The determination of nutrients followed the procedures applied in the measurement of the proximate composition of feeds.

### 2.5. Sample Collection and Chemical Composition Analysis of Abalone

Following the 24 h fasting period after the feeding experiment, all abalone from each cage were anesthetized with tricaine methanesulfonate (MS222) at a concentration of 100 mg/L, and then weighed collectively. Twenty abalone from each cage were randomly selected and frozen at −20°C to determine the proximate composition of the soft body. Before examination, abalone were slightly thawed, and the shell and soft body of the abalone were then separated. The shell length, width, and height of abalone were measured using a digital caliper in millimeters, and the ratio of soft body weight to total body weight was calculated to evaluate the index of nutritional status. Specific growth rate (SGR, %) = [(Ln final weight of abalone – Ln initial weight of abalone) × 100]/days of feeding (112 days).

The experimental feeds and carcasses of abalone were homogenized for the chemical analysis based on AOAC [32]. The same procedure and methods were used in Ji et al. [33]’s study.

### 2.6. Stress Resistance of Abalone Under Three Different Stressors

After measurement of the collective weight of abalone from each net, abalone were restocked in the same net cage. The same designed experimental feeds were supplied to abalone for 14 days to minimize the stress caused by the measurement of the weight of abalone. A total of 60 abalone from each net cage were randomly selected and assigned to three plastic containers (20 abalone per container) and then exposed to three different stressors (air, high temperature, and low salinity). Dead individuals were recorded and eliminated every 2 and 4 h during the stress exposure period and following the 5‐day monitoring period, respectively.

#### 2.6.1. Stress Resistance of Abalone Under Air Exposure

20‐one containers (20 abalone per container; three replicates per diet) were placed in a single 3‐ton plastic raceway tank. Seawater in the tank was totally discharged, and the abalone were exposed to air for 20 h. After that, the tank was refilled with underground seawater, followed by monitoring the survival of abalone for the next 5 days.

#### 2.6.2. Stress Resistance of Abalone Under High Temperature Exposure

20‐one containers (20 abalone per container; three replicates per diet) were placed in a single 3‐ton plastic raceway tank. Water temperature was elevated at a rate of 1°C per hour from 21 to 30°C by immersing a titanium heater (TH‐2000) with an autonomous thermostat. Abalone were maintained in 30°C seawater for 20 h, and seawater was then totally discharged. After that, the tank was replenished with underground seawater, followed by monitoring the survival of abalone for the next 5 days.

#### 2.6.3. Stress Resistance of Abalone Under Low Salinity Exposure

20‐one containers (20 abalone per container; three replicates per diet) were placed in a single 3‐ton plastic raceway tank. The salinity of seawater was decreased to 25 psu by blending with tap water and underground seawater (31 psu). YSI 6‐Series MultiParameter (YSI, Yellow Springs, OH, USA) was utilized to monitor salinity. Abalone were maintained in low‐saline water at 25 psu for 12 h, and the water was then totally discharged. After that, the tank was replenished with underground seawater, followed by monitoring the survival of abalone for the next 5 days.

### 2.7. Data Analysis

One‐way ANOVA and Turkey’s test were employed to compare the means among dietary treatments in SPSS 26.0 (SPSS Inc., Chicago, IL, USA). Water stability of feeds was determined by ANOVA with repeated measurement designs. Kaplan–Meier survival curve, Log‐rank, and Wilcoxon tests were conducted to evaluate the survival of abalone during the stress exposure and the 5‐day postobservation periods. All percentage data were arcsine‐transformed before analysis. Finally, regression analysis was performed by using survival of abalone under different types of stressors as the dependent variable and incorporating levels of CBC in feeds as the independent variable.

## 3. Results

### 3.1. Water Stability of Feeds

**Figure 1:**
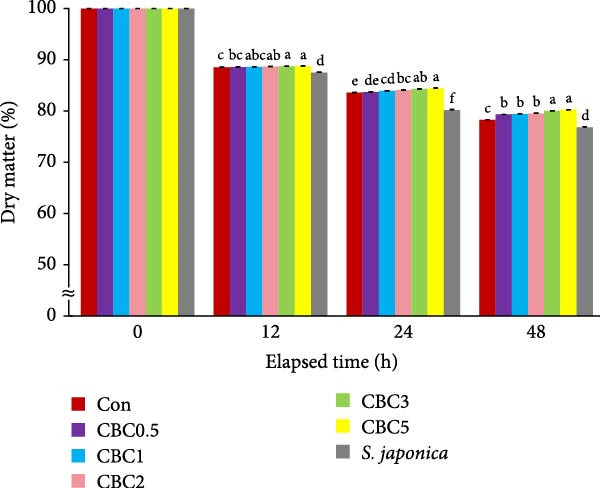
Changes in dry matter content (%) of the experimental diets following immersion in seawater for 12, 24, and 48 h (means of triplicate ± SE; ANOVA with repeated design: times [*p* < 0.0001] and the interaction of experimental diets and immersion time [*p* < 0.0001]). Different letters in each time point indicate significant (*p* < 0.05) differences between the experimental diets within each time point.

**Figure 2:**
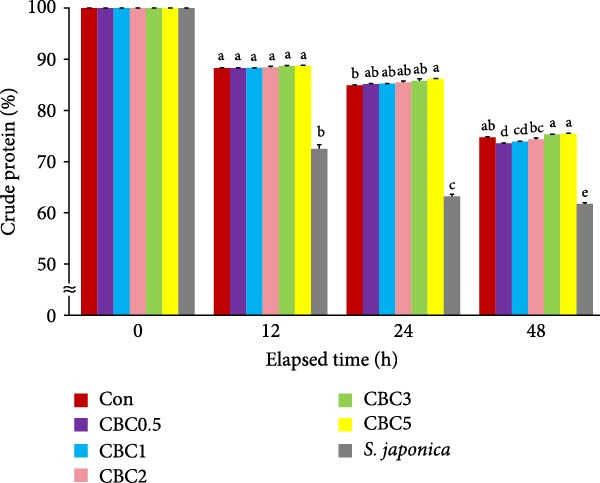
Changes in crude protein content (%) of the experimental diets following immersion in seawater for 12, 24, and 48 h (means of triplicate ± SE; ANOVA with repeated design: times [*p* < 0.0001] and the interaction of experimental diets and immersion time [*p* < 0.0001]). Different letters in each time point indicate significant (*p* < 0.05) differences between the experimental diets within each time point.

**Figure 3:**
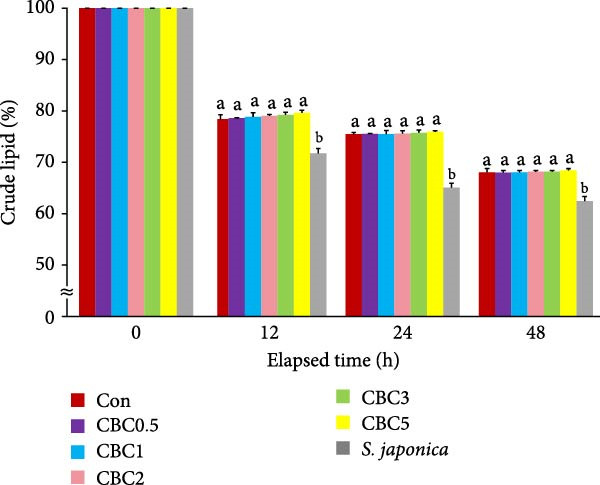
Changes in crude lipid content (%) of the experimental diets following immersion in seawater for 12, 24, and 48 h (means of triplicate ± SE; ANOVA with repeated design: times [*p* < 0.0001] and the interaction of experimental diets and immersion time [*p* < 0.0001]). Different letters in each time point indicate significant (*p* < 0.05) differences between the experimental diets within each time point.

**Figure 4:**
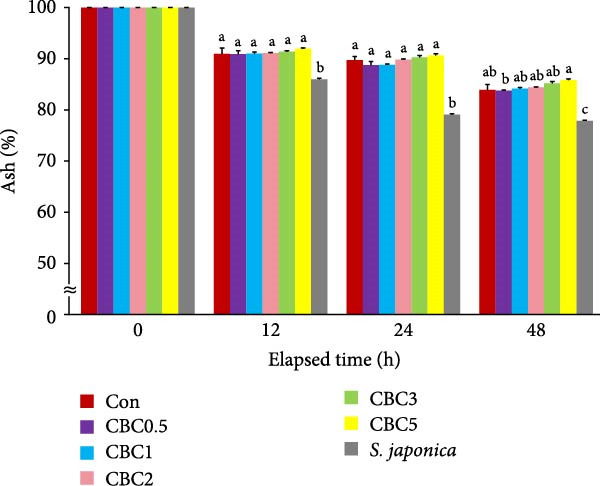
Changes in ash content (%) of the experimental diets following immersion in seawater for 12, 24, and 48 h (means of triplicate ± SE; ANOVA with repeated design: times [*p* < 0.0001] and the interaction of experimental diets and immersion time [*p* < 0.0001]). Different letters in each time point indicate significant (*p* < 0.05) differences between the experimental diets within each time point.

The content of dry matter (Figure 1), crude protein (Figure 2), crude lipid (Figure 3), and ash (Figure 4) of experimental feeds was statistically (*p* < 0.05 for all) influenced over all periods of time, and statistical (*p* < 0.05 for all) interactions between time and experimental feeds were also noticed. Throughout all periods of observation, the retention of dry matter content in all formulated feeds was statistically (*p* < 0.05) higher than in the *S. japonica*. At 12 h seawater immersion, the retained dry matter content in the CBC5 and CBC3 feeds was statistically (*p* < 0.05) greater than in the Con and CBC0.5 feeds, but not statistically different from the CBC1 and CBC2 feeds. However, at 24 and 48 h seawater immersion, the retained dry matter content of the CBC5 feed was statistically (*p* < 0.05) greater than in all formulated feeds, except for the CBC3 feed.

During every observational period, the retention of crude protein content in all formulated artificial feeds was statistically (*p* < 0.05) higher than in the *S. japonica*. At 24 h seawater immersion, the retained crude protein content in the CBC5 feed was statistically (*p* < 0.05) higher than in the Con feed, but not statistically different from all other formulated feeds. However, after 48 h seawater immersion, the retained crude protein in the CBC3 and CBC5 feeds was statistically (*p* < 0.05) greater than in the CBC0.5, CBC1, and CBC2 feeds, but not statistically different from the Con feed.

The retained crude lipid content in all formulated feeds was statistically (*p* < 0.05) greater than in the *S. japonica* throughout all observation periods. Nevertheless, no statistical difference in retained crude lipid content among the formulated feeds was observed during any observation period.

Throughout all observation periods, the retained ash content in all formulated feeds was statistically (*p* < 0.05) greater than in the *S. japonica*. After the 48 h seawater immersion, the retained ash content of the CBC5 feed was statistically (*p* < 0.05) greater than in the CBC0.5 feed, but not statistically different from all other formulated feeds.

### 3.2. Performance of Abalone

After the 16‐week feeding experiment, survival of abalone (75.56%–81.94%) was not statistically impacted by the incorporation of CBC in the feeds (Table 2). However, weight gain of abalone fed the CBC1, CBC2, CBC3, and CBC5 feeds was statistically (*p* < 0.05) greater than that of abalone fed *S. japonica*, but not statistically different from that of abalone fed the Con and CBC0.5 feeds. SGR of abalone fed all formulated feeds was statistically (*p* < 0.05) higher than that of abalone fed the *S. japonica*, but no significant difference in SGR was observed among all formulated feeds. Shell length, width, and height, and soft body weight of abalone fed all artificial feeds were statistically (*p* < 0.05 for all) greater than those of abalone fed the *S. japonica* (Table 3). The ratio of soft body weight to total weight of abalone was not statistically influenced by dietary treatments.

**Table 2:** Survival (%), weight gain (g/abalone), and specific growth rate (SGR, %/day) of juvenile Japanese abalone fed the experimental diets for 16 weeks.

Experimental diets	Initial weight (g/abalone)	Final weight (g/abalone)	Survival (%)	Weight gain (g/abalone)	SGR^1^ (%/day)
Con	3.33 ± 0.002	6.72 ± 0.01^ab^	78.61 ± 0.73	3.39 ± 0.01^ab^	0.63 ± 0.00^a^
CBC0.5	3.34 ± 0.001	6.74 ± 0.07^a^	78.06 ± 1.55	3.41 ± 0.07^ab^	0.63 ± 0.01^a^
CBC1	3.33 ± 0.002	6.79 ± 0.04^a^	81.94 ± 1.55	3.46 ± 0.04^a^	0.64 ± 0.01^a^
CBC2	3.33 ± 0.001	6.81 ± 0.12^a^	80.00 ± 4.59	3.47 ± 0.12^a^	0.64 ± 0.02^a^
CBC3	3.33 ± 0.002	6.85 ± 0.06^a^	80.00 ± 1.92	3.51 ± 0.06^a^	0.64 ± 0.01^a^
CBC5	3.33 ± 0.001	6.85 ± 0.06^a^	80.28 ± 1.94	3.51 ± 0.06^a^	0.64 ± 0.01^a^
*Saccharina japonica*	3.33 ± 0.001	6.39 ± 0.08^b^	75.56 ± 1.47	3.07 ± 0.08^b^	0.58 ± 0.01^b^
*p*‐Value	—	<0.007	>0.4	<0.008	<0.008

*Note:* Values (means of triplicate ± SE) in the same column with different superscript letters are significantly different (*p* < 0.05).

^1^Specific growth rate (SGR, %/day) = ([Ln final weight of abalone – Ln initial weight of abalone] × 100)/days of feeding.

**Table 3:** Shell length (mm), shell width (mm), shell height (mm), soft body weight (g), and the ratio of soft body weight to total weight of juvenile Japanese abalone fed the experimental diets for 16 weeks.

Experimental diets	Shell length (mm)	Shell width (mm)	Shell height (mm)	Soft body weight (g)	Soft body weight/total weight
Con	41.24 ± 0.20^a^	29.42 ± 0.14^a^	7.74 ± 0.02^a^	4.09 ± 0.06^a^	0.61 ± 0.01
CBC0.5	41.27 ± 0.11^a^	29.47 ± 0.03^a^	7.64 ± 0.03^a^	4.08 ± 0.05^a^	0.62 ± 0.01
CBC1	41.30 ± 0.07^a^	29.45 ± 0.03^a^	7.67 ± 0.02^a^	4.04 ± 0.05^a^	0.61 ± 0.01
CBC2	40.90 ± 0.34^a^	29.51 ± 0.03^a^	7.69 ± 0.03^a^	4.04 ± 0.04^a^	0.61 ± 0.01
CBC3	41.23 ± 0.05^a^	29.50 ± 0.03^a^	7.64 ± 0.04^a^	4.11 ± 0.03^a^	0.62 ± 0.01
CBC5	41.26 ± 0.07^a^	29.47 ± 0.07^a^	7.69 ± 0.04^a^	4.14 ± 0.02^a^	0.62 ± 0.00
*Saccharina japonica*	39.27 ± 0.13^b^	27.37 ± 0.13^b^	7.08 ± 0.03^b^	3.81 ± 0.03^b^	0.60 ± 0.01
*p*‐Value	<0.001	<0.001	<0.001	<0.001	>0.3

*Note:* Values (means of triplicate ± SE) in the same column with different superscript letters are significantly different (*p* < 0.05).

### 3.3. Chemical Composition of the Soft Body of Abalone

The moisture (77.43%–77.99%), crude protein (15.80%–16.37%), and crude lipid (1.09%–1.14%) content of the soft body of abalone were not statistically influenced by inclusion of CBC in diets (Table 4). The ash content of the soft body of abalone fed the CBC3 feed was statistically (*p* < 0.05) greater than that of abalone fed all other formulated feeds, but not statistically different from that of abalone fed the *S. japonica*.

**Table 4:** Chemical composition (%, wet weight basis) of the soft body of Japanese abalone at the end of the 16‐week feeding trial.

Experimental diets	Moisture	Crude protein	Crude lipid	Ash
Con	77.96 ± 0.52	16.25 ± 0.32	1.10 ± 0.03	2.22 ± 0.01^d^
CBC0.5	77.82 ± 0.19	16.03 ± 0.31	1.13 ± 0.02	2.45 ± 0.03^bc^
CBC1	77.67 ± 0.58	15.80 ± 0.22	1.10 ± 0.02	2.33 ± 0.04^cd^
CBC2	77.86 ± 0.22	15.97 ± 0.25	1.09 ± 0.01	2.43 ± 0.04^bc^
CBC3	77.99 ± 0.15	16.35 ± 0.09	1.10 ± 0.02	2.64 ± 0.02^a^
CBC5	77.46 ± 0.11	16.21 ± 0.17	1.14 ± 0.01	2.50 ± 0.02^bc^
*Saccharina japonica*	77.43 ± 0.23	16.37 ± 0.30	1.10 ± 0.01	2.59 ± 0.01^ab^
*p*‐Value	>0.6	>0.8	>0.4	<0.001

*Note:* Values (means of triplicate ± SE) in the same column with different superscript letters are significantly different (*p* < 0.05).

### 3.4. Survival of Abalone Under Various Stressors

#### 3.4.1. Survival of Abalone Under Air Exposure

**Figure 5:**
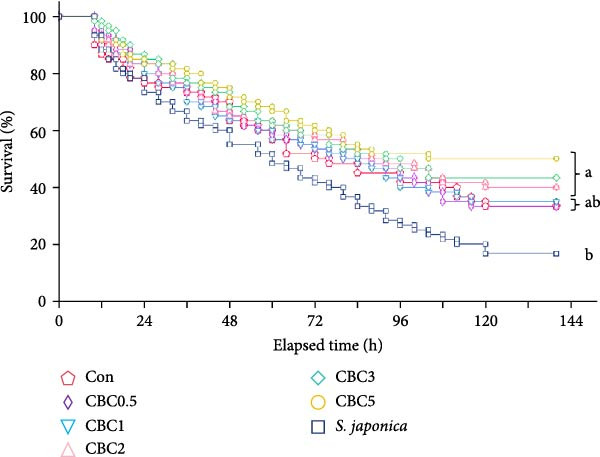
Survival (%) of Japanese abalone fed the experimental diets with the supplementation of combined broccoli by‐product and citrus by‐product meal for 16 weeks, and then subjected to 20 h air exposure for the next 5 days (means of triplicate ± SE; different letters indicate significance at *p* < 0.05 for log‐rank and Wilcoxon tests; regression analysis; *Y* [survival of abalone] = 3.5593 × [incorporated CBC levels in diets] + 32.3446, *p* < 0.0001, *R* ^2^ = 0.7583).

Mortality of abalone was noticed at 10 h after the initiation of the air exposure (Figure 5). After the 5‐day observation phase, survival of abalone fed the CBC5 (50.0%), CBC3 (43.3%), and CBC2 (40.0%) feeds was statistically greater than that of abalone fed the *S. japonica* (16.7%), but not statistically different from that of abalone fed the CBC1 (35.0%), CBC0.5 (33.3%), and Con (33.3%) feeds. Survival of abalone linearly increased with increasing inclusion levels of CBC in feeds (*Y* [survival of abalone] = 3.5593*X* [inclusion levels of CBC in feeds] + 32.3446, *p* < 0.0001, *R* ^2^ = 0.7583) after the 5‐day observation phase, according to regression analysis.

#### 3.4.2. Survival of Abalone Under High Temperature Exposure

**Figure 6:**
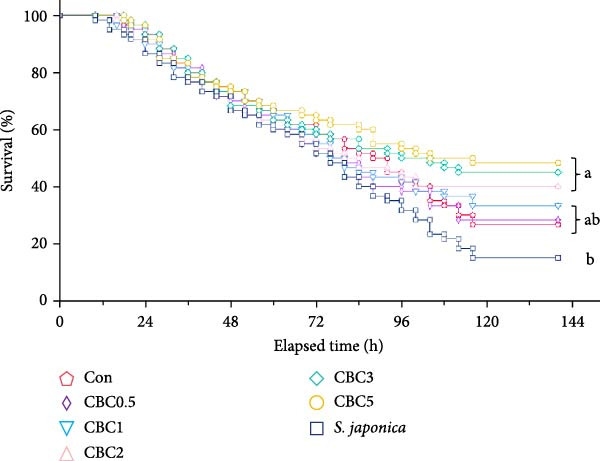
Survival (%) of Japanese abalone fed the experimental diets with the supplementation of combined broccoli by‐product and citrus by‐product meal (CBC) for 16 weeks, and then subjected to 20 h high temperature at 30°C exposure for the next 5 days (means of triplicate ± SE; different letters indicate significance at *p* < 0.04 for log‐rank and Wilcoxon tests; regression analysis; *Y* [survival of abalone] = 4.6085 × [incorporated CBC levels in diets] + 28.1114, *p* < 0.0001, *R* ^2^ = 0.8080).

Mortality of abalone was observed at 10 h after the initiation of the high temperature exposure (Figure 6). After the 5‐day observation phase, survival of abalone fed the CBC5 (48.3%), CBC3 (45.0%), and CBC2 (40.0%) feeds were statistically (*p* < 0.05) greater than that of abalone fed the *S. japonica* (15.0%), but not statistically different from that of abalone fed the CBC1 (33.3%), CBC0.5 (28.3%), and Con (26.7%) feeds. Survival of abalone linearly increased with increasing inclusion levels of CBC in feeds (*Y* [survival of abalone] = 4.6085 *X* [inclusion levels of CBC in feeds] + 28.1114, *p* < 0.0001, *R* ^2^ = 0.8080) after the 5‐day observation phase, according to regression analysis.

#### 3.4.3. Survival of Abalone Under Low Salinity Exposure

**Figure 7:**
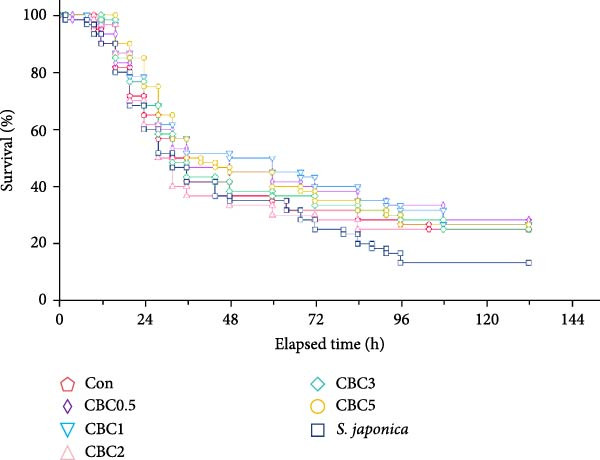
Survival (%) of Japanese abalone fed the experimental diets with the supplementation of combined broccoli by‐product and citrus by‐product meal (CBC) for 16 weeks, and then subjected to 12 h low salinity at 25 psu exposure for the next 5 days (means of triplicate ± SE).

Mortality of abalone was observed at 2 h after the initiation of the low salinity exposure (Figure 7). However, by the end of the 5‐day postobservation period, survival of abalone varied from 13.3% to 28.3%, with no statistical difference among dietary treatments.

## 4. Discussion

The comparable SGR values of abalone achieved in the current experiment to those reported for abalone of similar size (3–6 g) in other studies [3, 10, 33] demonstrated that the growing performance of abalone was satisfactory. Additionally, greater SGR values of abalone fed all formulated artificial feeds versus abalone fed the *S. japonica* align with the results of prior investigations [3, 10, 11, 13, 14], indicating that the nutritionally‐balanced formulated feeds may improve the growth of abalone over MA. Our previous studies [3, 10] also proved that abalone achieved excellent growth performance and stress reduction when CPB and BBP were included as functional additives and used as substitutes for *S. japonica* in abalone feeds, respectively. Thus, CPB and BBP could be considered natural growth enhancers or stress reducers in abalone feeds. However, the effect of combined natural plant additives on the growth and stress resistance of abalone has not been evaluated.

Although the current research did not establish a statistical relationship between CBC incorporating levels and the growth of abalone, a slightly elevated trend of weight gain and SGR was noticed in abalone fed feeds containing 0.5%–3% CBC. Similarly, Dai and Cho [10] also revealed that the weight gain and SGR of abalone were linearly improved with increasing incorporation levels of CPB in feeds. Li et al. [3] also demonstrated that the growth performance was not negatively affected by the substitution of *S. japonica* by BBP in diets when abalone were supplied with feeds replacing 25%–100% of *S. japonica* by BBP. The growth performance of abalone in this study and our previous studies indicated that incorporation of single or combined agricultural by‐products within a specific range would not negatively influence the growth performance of abalone.

Shell growth measurements, including shell length, width, height, and the soft body weight of abalone fed all artificial feeds, were superior to abalone fed the *S. japonica* in this experiment. However, these measurements were not impacted by the incorporation ratios of CBC in feeds, indicating that the functional additive, CBC, had no desirable impact on the shell growth or soft body weight of abalone. However, several studies [33–35] have shown that the shell growth measurements and soft body weight are generally consistent with the overall growth of abalone.

The higher leaching rate of *S. japonica* compared to the formulated feeds in the current experiment indicated that the formulated feeds have relatively high stability in seawater. Additionally, the lower leaching rates of crude protein, crude lipid, dry matter, and ash in all artificial feeds compared to *S. japonica* after 12, 24, and 48 h of seawater immersion could be one of the key factors contributing to the relatively good growth of abalone fed the formers. The leaching rates of dry matter, crude protein, lipid, and ash tended to increase with immersion time. Similarly, both diets and immersion time significantly influenced the carbon:nitrogen (C:N) ratio of feeds in tanks, regardless of the presence of abalone [36]. Additionally, the interaction between diet and immersion time exhibited a pronounced effect on phosphorus (P) levels and the C: N ratio of feeds in tanks without abalone. After the 48 h seawater immersion, the greatest retention of dry matter, crude protein, and ash was found in the CBC5 feed, which aligns with the findings that abalone fed the CBC5 feed showed the greatest weight gain in the current study. Pectin, which is abundant in citrus peels, contributes thickness, emulsification, and stabilizing properties to various foods, such as jams, jellies, and candies [37]. In the current experiment, the inclusion of CBC in feeds tended to enhance the water stability of feeds, possibly due to the presence of pectin in CPB.

The moisture, crude protein, and crude lipid content of the carcass of abalone were unaffected by the inclusion levels of CBC in diets in this experiment, which was consistent with our previous research [10] showing that the soft body chemical composition of abalone was not impacted by the dietary inclusion levels of CPB. Similarly, the chemical composition of the carcass of abalone was not altered by the dietary inclusion of herbs [38], antimeal (coproduct of the wine industry) [39], and squid meal [38]. However, the ash content of the chemical composition of abalone was influenced by dietary treatments, which aligns with the results of a previous study [3] showing that the ash content of abalone was altered by dietary replacements of MA (*S. japonica*) with BBP. Likewise, Ju et al. [40] demonstrated that the ash content in the soft body of abalone was altered by supplementation of 3% each of three species of algae or 0.02% pigment extract from the abalone shells in diets. The discrepancy in the results in these studies could be attributed to the different types and concentrations of additives used in abalone feeds.

Mollusks are often inevitably exposed to air because of essential handling procedures, including long‐distance transportation and size grading [22]. Although abalone could maintain basic metabolic activities at a decreased rate during air exposure [41], high mortality might occur due to their limited capacity to tolerate these stressors. The high mortality rate of abalone could be attributed to fluctuations in immune responses and the transcription of specific genes during handling or air exposure [42]. Similarly, air exposure could affect the immune functions of scallop (*Chlamys farreri*) hemocytes, with high‐temperature air exposure leading to elevated mortality in scallops [43]. In this experiment, following the 5‐day air exposure trial, abalone fed the CBC5, CBC3, and CBC2 diets showed higher survival than those fed the *S. japonica*. Moreover, survival increased linearly with the dietary inclusion of CBC, indicating that CBC supplementation enhances abalone’s tolerance to air exposure stress. Thus, incorporating at least 2% CBC into abalone diets is recommended during long‐distance transportation or size grading in aquaculture operations. Similarly, a linear relationship between the incorporation level of CPB in formulated feeds and the survival rate of abalone after the 5‐day observation phase of 20‐h air exposure was also reported [10].

In summer, high temperatures of seawater are one of the most serious environmental issues in the abalone culture industry and is commonly related to reduced immunity and antioxidative capacity and outbreaks of infectious disease [4, 11, 22, 44]. According to Lei et al. [45], during heat stress, the excess dietary eicosapentaenoic acid could increase the falling rate of abalone, which was adhering to a polyethylene plate. They clarified that the reduced antioxidative activity and increased threat of inflammation could be the main reasons for the rapid falling of abalone. Ma et al. [4] indicated that the incorporation of chromium yeast (CrYst, 2 mg/kg) and astaxanthin (ASTA, 80 mg/kg), or a combination of CrYst and ASTA in diets, significantly reduced the falling rate of abalone during heat stress, and combined CrYst and ASTA exhibited a lower cumulative mortality compared to using them individually after a 16‐h heat stress test. After the 5‐day observation period following the 20 h exposure to high temperature (30°C) in this experiment, the survival of abalone fed the CBC5, CBC3, and CBC2 feeds was greater than that of abalone fed the *S. japonica*. Moreover, the survival of abalone increased linearly with the higher CBC levels in diets, indicating that abalone fed with high CBC levels (2%, 3%, and 5%) had an enhanced ability to tolerate high temperature stress. Therefore, incorporating at least 2% CBC into diets represents a practical strategy for abalone farmers to enhance abalone’s tolerance to high summer temperature. Similarly, inclusion of CPB [10] or grape seed extract [11] in formulated feeds has been shown to improve abalone tolerance to elevated temperature.

During the events of severe summer rainstorms and typhoons, the salinity of seawater in abalone farms may reduce to around 25 psu, and the climate deterioration in recent years is more likely to exacerbate this situation [21, 46]. Abalone is extremely sensitive to low salinity [47], and most mollusks adjust to salinity stress by restoring inorganic and organic ionic balance [48]. According to Boamah et al. [21], reduced salinity could impact the heart rate of abalone, and hybridization might be one of the potential ways to produce more stress‐resistant abalone. However, after the 5‐day observation phase following the 12 h exposure to low salinity at 25 psu, the survival of abalone fed all feeds was no more than 29%, and it was not influenced by dietary treatments. Likewise, the incorporation of CPB in feeds did not influence the capacity of abalone to tolerate the low salinity at 25 psu [10]. This may be due to the detrimental impact of low salinity on abalone survival, which cannot be significantly alleviated by limited doses (≤5%) of natural additives alone. Although no significant difference in survival was observed among abalone fed all diets during the 5‐day postobservation period following the 12 h low salinity exposure, a slight improvement in survival was noticed in abalone receiving the formulated diets compared to abalone receiving *S. japonica*. This aligns with findings by Boarder and Shpigel [49], who reported that juvenile abalone (*H. roei*) supplied with protein‐enriched *Ulva rigida* exhibited 0% survival, whereas abalone fed formulated feeds achieved over 40% survival rate after the 96 h exposure to 20 g/L salinity. However, unlike this study, applying thraustochytrid (*Schizochytrium* sp.) as a lipid source in diets could reduce the mortality in abalone (*H. asinina*) following the 96 h salinity stress at 20 g/L [50].

The capacity of abalone to withstand high temperature and air exposure stressors significantly increased when the dietary inclusion level of CBC was at least 2%, compared to abalone fed the *S. japonica*. This improvement may be due to the bioactive substances in CPB and BBP included in diets. Citrus peels, which make up about 50% of the wet fruit weight, are rich in phenolic compounds, including phenolic acids and flavonoids [19, 51]. In China, dried citrus peel is traditionally used as an herbal medicine for humans, and herbal extracts can also serve as functional additives in aquafeeds due to their innumerable beneficial effects [10, 52]. Hosseini et al. [53] demonstrated that incorporating pectin derived from orange peels into feed could improve growth and feed efficiency of common carp (*C. carpio*). Additionally, the inclusion of 1% pectin derived from orange peels in diets enhanced skin mucus lysozyme and serum peroxidase activities in the fish. BBP are rich in vitamins, β‐carotene, minerals, and total phenolic content, and they exhibit high antioxidant and anticancer activities [3, 18]. According to Hwang and Lim [18], broccoli leaf extracts exhibited greater cell growth inhibitory activity on human lung carcinoma cells than the floret extracts. Although the growth of abalone was unaffected by dietary incorporation of CBC, the capacity of abalone to withstand high temperature and air exposure was significantly improved. This enhancement may be because of the high content of bioactive compounds in CPB and BBP, which possess antioxidant, anti‐inflammatory, anticarcinogenic, and antibacterial properties. These compounds likely have a greater impact on enhancing immune response in abalone. Similarly, Lee et al. [54] reported that the weight gain and feed utilization of whiteleg shrimp (*Litopenaeus vannamei*) were not influenced by incorporating 1%–3% fermented lemon peel into their diets. However, the inclusion of 2% fermented lemon peel significantly improved the total hemocyte count, phenoloxidase activity, and survival rate of whiteleg shrimp during the 72 h postobservation period after the infection of *Vibrio alginolyticus*. Several studies have demonstrated that supplementation of BBP or CPB in diets could improve the immune responses of abalone [3, 10], gilthead seabream (*Sparus aurata*) [55], rainbow trout (*Oncorhynchus mykiss*) [56], and rohu (*Labeo rohita*) [57]. Further studies should be conducted in the future to investigate the impacts of specific bioactive compounds in CPB and BBP on the growth and health status of abalone to clarify the unresolved mechanisms in this study.

## 5. Conclusion

The combined agricultural by‐product of CBC could be used as a potential natural antioxidant additive and stress reducer in abalone diets. Incorporating at least 2% CBC into diets is recommended to enhance abalone’s capability to withstand air and high temperature stressors. However, the specific bioactive compounds that enhance abalone performance, as well as gene expression under various dietary conditions, need to be further investigated in future studies.

## Conflicts of Interest

The authors declare no conflicts of interest.

## Author Contributions

**Ran Li:** investigation, data curation, methodology, writing – original draft. **Sung Hwoan Cho:** conceptualization, project administration, methodology, writing – review and editing.

## Funding

This research was supported by the Basic Science Research Program through the National Research Foundation of Korea (NRF), funded by the Ministry of Science, ICT and Future Planning (Grant 2017R1A2B4009773). This work was supported by the National Research Foundation of Korea (NRF) grant funded by the Korean Government (MSIT) (Grant 2020R1A2C1009903).

## Data Availability

The data that support the findings of this study are available from the corresponding author upon reasonable request.
